# A Case of Appendiceal Endometriosis Diagnosed as Acute Appendicitis and Treated With Laparoscopic Appendectomy

**DOI:** 10.7759/cureus.96409

**Published:** 2025-11-09

**Authors:** Tomonari Shimagaki, Naotaka Hashimoto, Takuro Kometani, Kenkichi Hashimoto, Takashi Maeda

**Affiliations:** 1 Department of Surgery, Hiroshima Red Cross Hospital and Atomic-bomb Survivors Hospital, Hiroshima, JPN

**Keywords:** acute appendicitis, appendiceal endometriosis, estrogen receptor (er), laparoscopic appendectomy, laparoscopic surgery for endometriosis

## Abstract

Appendiceal endometriosis is an uncommon manifestation of extrapelvic endometriosis that can clinically and radiologically mimic acute appendicitis. We report the case of a 41-year-old woman who presented with acute right lower quadrant abdominal pain and laboratory findings suggestive of appendicitis. Laparoscopic appendectomy was performed, and histopathology revealed endometrial glands and stroma within the muscularis propria and mesoappendiceal fat, consistent with appendiceal endometriosis. The postoperative course was uneventful, and the patient remained symptom-free at follow-up. This case underscores the necessity of considering appendiceal endometriosis as a rare but potential differential diagnosis of acute abdomen in women of reproductive age and highlights the pivotal role of surgical intervention in both establishing the diagnosis and providing definitive treatment.

## Introduction

Endometriosis is an estrogen-dependent condition characterized by the ectopic implantation of endometrial glands and stroma outside the uterine cavity, typically accompanied by a chronic inflammatory response [1]. Endometriosis affects 6 to 10% of women of reproductive age and can manifest in several locations [2]. The clinical manifestations of endometriosis vary according to the anatomical location of the lesions. While acute appendicitis remains the most frequent surgical cause of right lower quadrant abdominal pain [3], appendiceal endometriosis represents an exceptionally uncommon form of extragonadal endometriosis. This condition primarily occurs in women of reproductive age and may present with a diverse range of symptoms that can mimic acute appendicitis or other intra-abdominal pathologies [4]. Here, we report a case of appendiceal endometriosis presenting as acute right lower quadrant abdominal pain and highlight its relevance as a rare differential diagnosis of acute appendicitis in women of childbearing age.

## Case presentation

A 41-year-old woman presented with right lower abdominal pain of two days’ duration. The pain was intermittent and initially tolerable, but worsening prompted evaluation at a local clinic, where ultrasonography suggested appendicitis or diverticulitis. There were no cyclical symptoms. She was subsequently referred to our hospital. She reported daily bowel movements, denied pregnancy, and noted that her last menstrual period had occurred a few days earlier. Obstetric history was gravida 1, para 1. Past medical history was notable for rheumatoid arthritis. She had no allergies, took no regular medications, and reported no smoking and occasional alcohol consumption. Her medical history did not reveal any dysmenorrhea or chronic pelvic pain.

On admission, her general condition was stable. Vital signs were blood pressure 115/74 mmHg, pulse 88 bpm, and body temperature 37.0°C. Abdominal examination showed a flat, soft abdomen with localized tenderness in the right lower quadrant, accompanied by mild rebound tenderness without guarding. Laboratory studies demonstrated a white blood cell count of 7,400/μL and elevated C-reactive protein at 9.77 mg/dL. All the laboratory tests are shown in Table 1.

**Table 1:** Laboratory findings upon admission. WBC: White Blood Cells, RBC: Red Blood Cells, Hb: Hemoglobin, Hct: Hematocrit, Plt: Platelets, PT-INR: Prothrombin Time-International Normalized Ratio, APTT: Activated Partial Thromboplastin Time, PT (%): Prothrombin Time (Percentage), TP: Total Protein, Alb: Albumin, T.Bil: Total Bilirubin, D.Bil: Direct Bilirubin,  BUN: Blood Urea Nitrogen, Cr: Creatinine, AST: Aspartate Aminotransferase, ALT: Alanine Aminotransferase, LDH: Lactate Dehydrogenase, CRP: C-Reactive Protein

Peripheral blood			(reference range)
WBC	7400	/μl	(3300-8600)
RBC	4.09x10^6^	/μl	(3.86-4.92)
Hb	11.6	g/dl	(11.6-14.8)
Hct	36.5	%	(35.1-44.4)
Plt	21.3x10^4^	/μl	(15.8-34.8)
Coagulation			
PT-INR	1.16		
APTT	39.5	sec	(25.1-37.4)
PT(%)	64.9	%	(80.0-120.0)
Biochemistry			
TP	6.9	mg/dl	(6.6-8.1)
Alb	3.8	g/dl	(4.1-5.1)
T.Bil	0.6	mg/dl	(0.4-1.5)
D.Bil	0.1	mg/dl	(0.0-0.3)
BUN	8.5	mg/dl	(8.0-20.0)
Cr	0.49	mg/dl	(0.46-0.79)
AST	16	U/L	(13-30)
ALT	14	U/L	(9-32)
LDH	156	U/L	(124-222)
CRP	9.77	mg/dl	(0.0-0.14)

**Figure 1:**
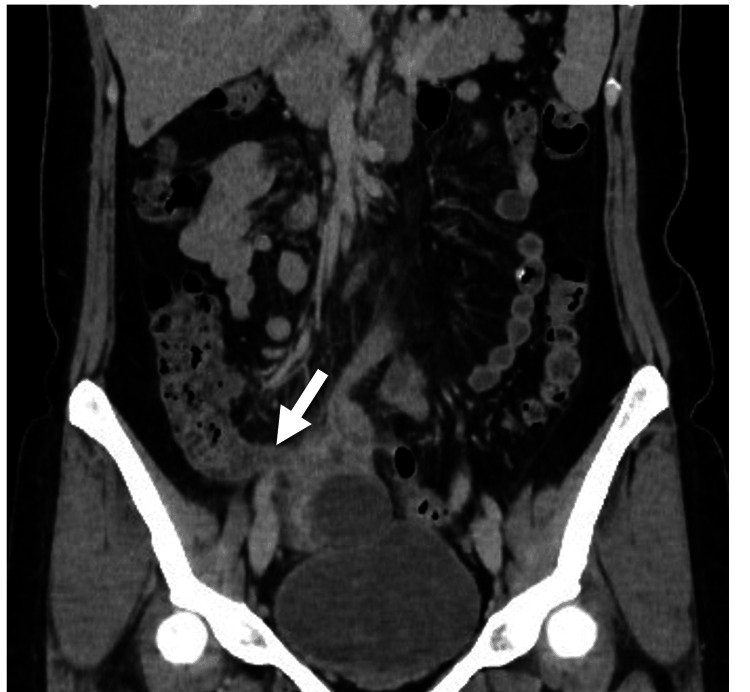
Contrast-enhanced axial abdominal CT revealed localized inflammatory changes in the mesenteric fat adjacent to the ileocecal junction. The appendix was enlarged with wall thickening (arrow).

Contrast-enhanced abdominal computed tomography (CT) revealed localized inflammatory changes in mesenteric fat adjacent to the ileocecal region. The appendix was enlarged to 8 mm with wall thickening but no intraluminal fluid (Figure 1). No appendicoliths were observed. The tip demonstrated indistinct margins with contrast-enhancing soft tissue, raising concern for a post-perforation state. Surrounding small bowel loops were thickened and adherent to the appendix. No ileus or free air was observed (Figure 1). A right ovarian cyst measuring 63 × 43 mm and a small volume of pelvic ascites were also noted. Based on these findings, acute appendicitis was diagnosed, and emergency surgery was performed.

**Figure 2:**
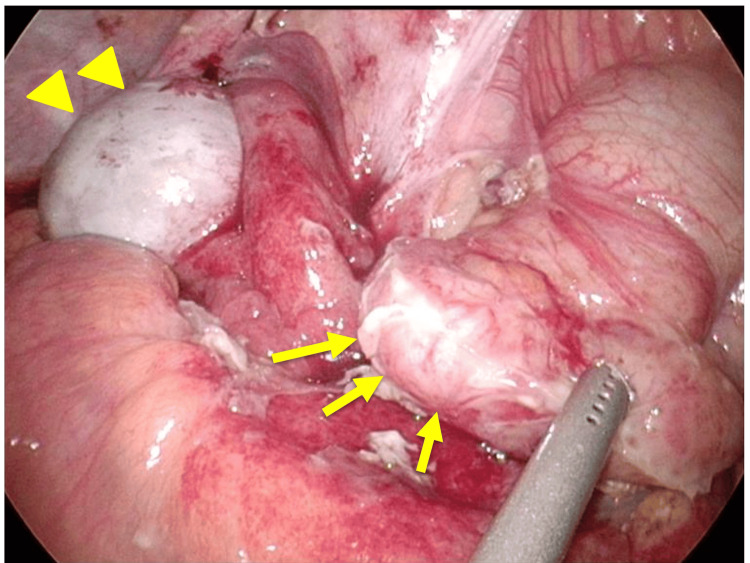
Intraoperative findings. An enlarged appendix was identified adherent to surrounding tissues such as the ileum, without evidence of perforation (arrow). A right ovarian cyst was also identified (arrowhead).

**Figure 3:**
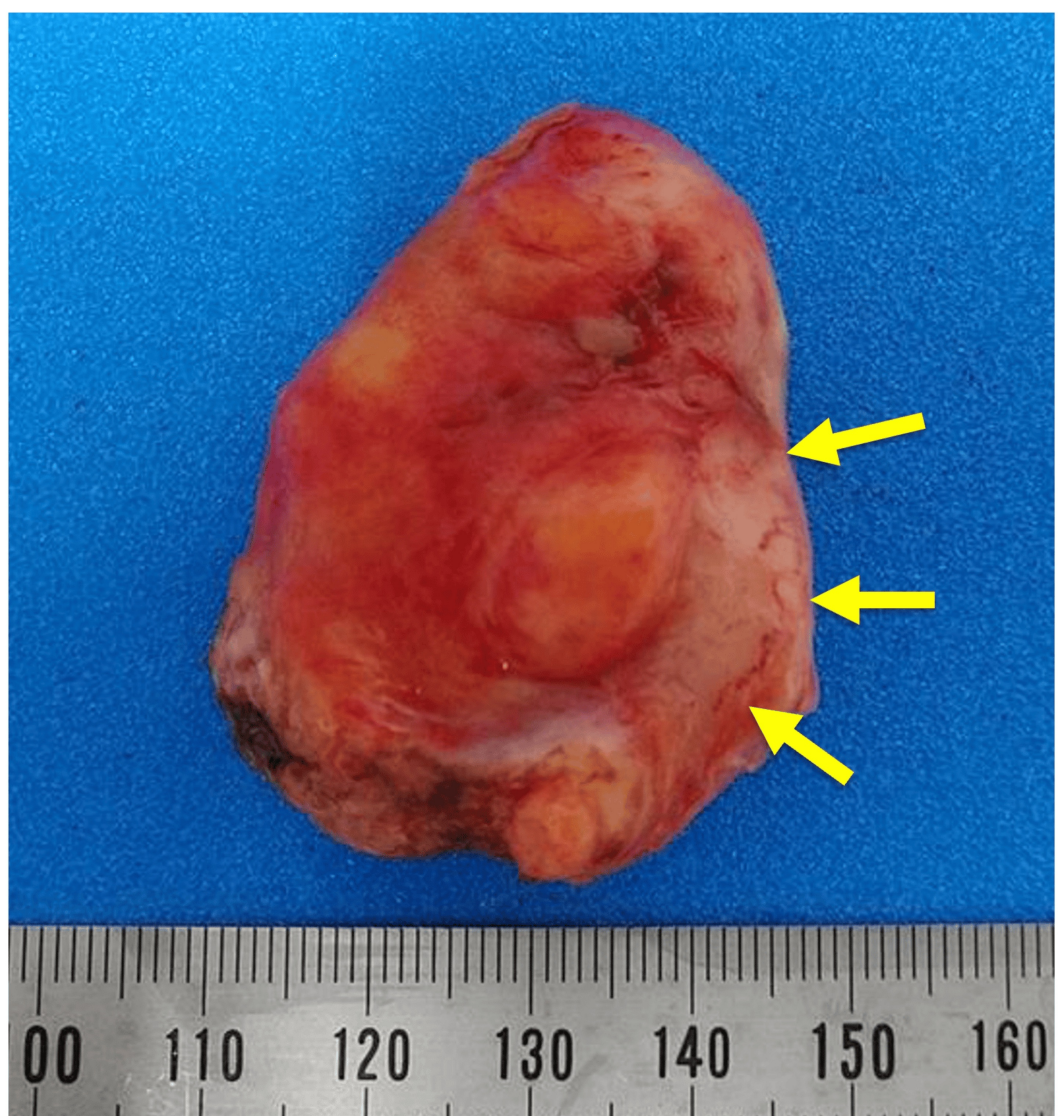
Macroscopic findings of the surgical specimen of the enlarged appendix (arrow).

Laparoscopic exploration revealed adhesions around the ileum and ascending colon. Blunt dissection exposed a markedly thickened appendix without evidence of perforation (Figure 2). The mesoappendix and appendiceal artery were divided, and the appendix was transected after ligation at the base with an Endoloop. The resected specimen demonstrated diffuse wall thickening and edema without tumor formation (Figure 3). The pelvic laparoscopic findings included a small amount of ascites, a normal uterus, and a right ovarian cyst, which was incidentally noted. The patient’s postoperative recovery was uneventful, and she was discharged on postoperative day three.

**Figure 4:**
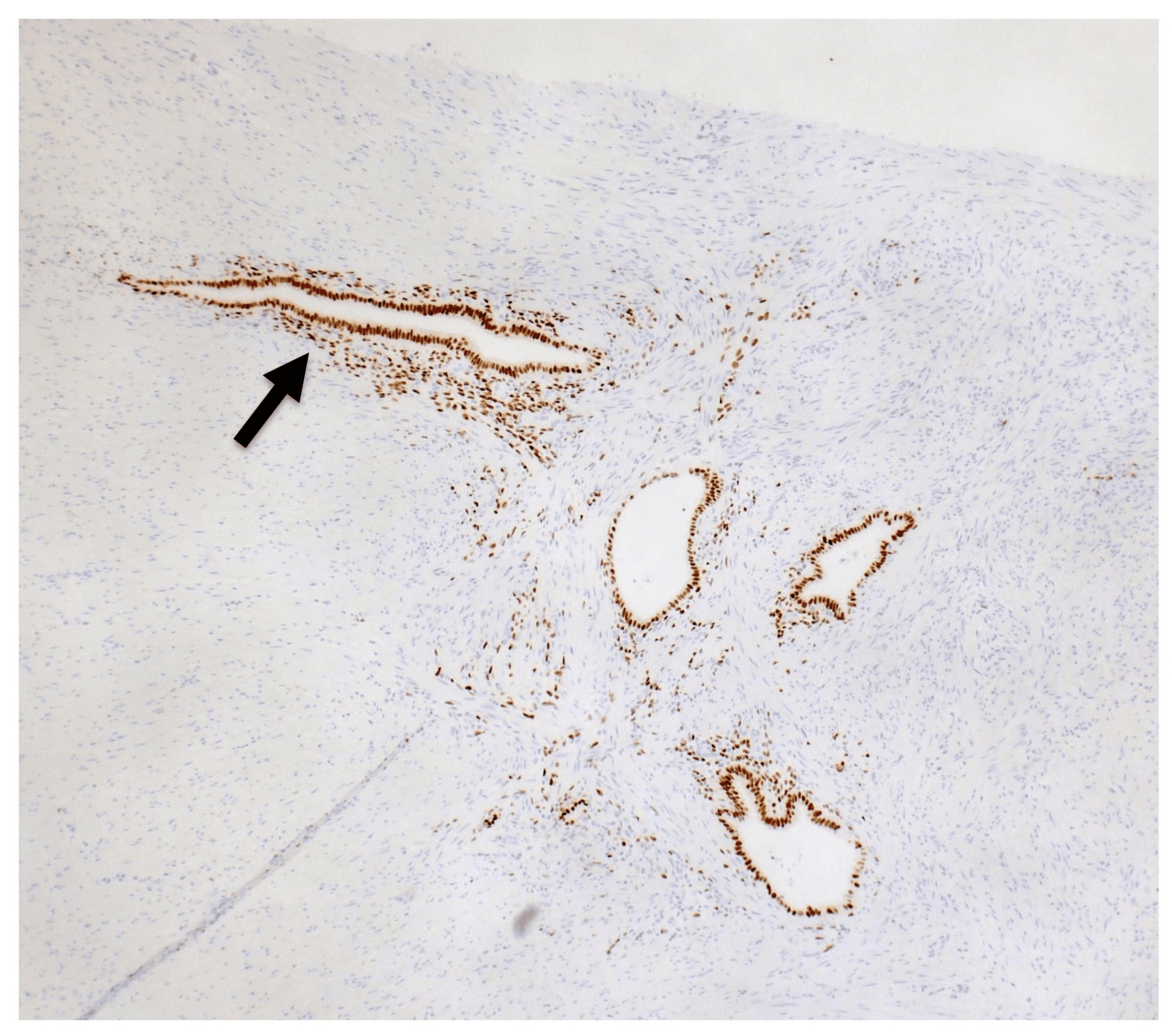
Microscopic findings: Endometrial stroma surrounding cystically dilated glands exhibiting positive immunoreactivity for the estrogen receptor (arrow). Immunohistochemical staining, ×40 magnification.

Histopathological analysis revealed non-neoplastic glandular structures within the muscularis propria and mesoappendiceal fat, surrounded by endometrial stroma, consistent with endometriosis. The mucosa demonstrated only chronic inflammatory cells without active inflammation. Abscess formation, fat necrosis, and fibrosis were present within the mesoappendiceal fat. Immunohistochemistry for p16, MDM2, and MIB-1 showed no features of malignancy. Estrogen receptor positivity was observed in the glandular epithelium and surrounding stroma (Figure 4). At three-month and six-month follow-up, including gynecological evaluation, the patient remained asymptomatic with no postoperative complications.

## Discussion

Endometriosis is a chronic, estrogen-dependent disorder characterized by the presence of endometrial glands and stroma outside the uterine cavity. Although commonly affecting the ovaries and pelvic peritoneum, extrapelvic sites such as the gastrointestinal tract are occasionally involved. Among these, the appendix is an uncommon location, with reported prevalence ranging from 0.05% to 0.8% in appendectomy specimens [5,6]. The present case illustrates the diagnostic challenge of appendiceal endometriosis, which can mimic acute appendicitis both clinically and radiologically.

Although acute appendicitis remains the most common etiology of right lower quadrant abdominal pain, appendiceal endometriosis should be considered a rare but important differential diagnosis in women of reproductive age. The clinical presentation is often nonspecific, characterized by symptoms such as abdominal pain, nausea, and alterations in bowel habits, which may overlap with those of various gynecologic disorders [7]. In this case, the patient presented with acute right lower quadrant tenderness and elevated inflammatory markers, consistent with appendicitis. Imaging revealed an enlarged appendix with periappendiceal inflammation, further reinforcing this impression. However, the absence of luminal obstruction and the presence of an ovarian cyst could have raised consideration for endometriosis. Given the acute presentation, however, surgical intervention was appropriately prioritized.

Histopathology confirmed the diagnosis by demonstrating ectopic endometrial glands and stroma within the muscularis propria and mesoappendiceal fat. The lack of active mucosal inflammation, despite clinical features suggestive of appendicitis, suggests that the patient’s symptoms were more likely attributable to endometriotic infiltration and associated chronic inflammation. Abscess formation, fat necrosis, and fibrosis observed in the mesoappendix are consistent with recurrent cyclical bleeding and inflammatory response, which are hallmarks of the disease process [8]. This underscores the importance of routine histopathological evaluation of all appendectomy specimens, as macroscopic findings alone are frequently nonspecific.

Laparoscopic appendectomy is both diagnostic and therapeutic in such cases. The minimally invasive approach enables the removal of the appendix while allowing inspection of pelvic structures for additional endometriotic lesions. In the present patient, surgery resolved acute symptoms and confirmed the diagnosis without postoperative complications. Although appendectomy is curative for appendiceal involvement, long-term management of endometriosis may require gynecological evaluation and, in selected cases, hormonal therapy to prevent recurrence [6]. Multidisciplinary follow-up is therefore recommended to optimize outcomes.

The present case highlights several important clinical lessons. First, appendiceal endometriosis, although rare, should be considered in the differential diagnosis of right lower quadrant pain in women of childbearing age, particularly when gynecological findings such as ovarian cysts coexist [6]. Second, histopathological analysis remains essential, as clinical and imaging findings are insufficient for definitive diagnosis. Finally, close collaboration between surgeons, pathologists, and gynecologists ensures appropriate diagnosis, treatment, and long-term follow-up for women with extrapelvic manifestations of endometriosis [9].

## Conclusions

Appendiceal endometriosis is a rare but important mimic of acute appendicitis. Awareness of this entity is essential for timely recognition and appropriate management. Although surgical removal is curative for localized appendiceal disease, comprehensive care should include evaluation for systemic endometriosis to guide long-term treatment strategies.
